# Publisher Correction: Frequent new particle formation over the high Arctic pack ice by enhanced iodine emissions

**DOI:** 10.1038/s41467-020-19533-y

**Published:** 2020-10-28

**Authors:** Andrea Baccarini, Linn Karlsson, Josef Dommen, Patrick Duplessis, Jutta Vüllers, Ian M. Brooks, Alfonso Saiz-Lopez, Matthew Salter, Michael Tjernström, Urs Baltensperger, Paul Zieger, Julia Schmale

**Affiliations:** 1 Laboratory of Atmospheric Chemistry, Paul Scherrer Institute, Villigen, PSI Switzerland; 2 Department of Environmental Science & Bolin Centre for Climate Research, Stockholm University, Stockholm, Sweden; 3 Department of Physics and Atmospheric Science, Dalhousie University, Halifax, Canada; 4 School of Earth and Environment, University of Leeds, Leeds, UK; 5 Department of Atmospheric Chemistry and Climate, Institute of Physical Chemistry Rocasolano, CSIC, Madrid, Spain; 6 Department of Meteorology & Bolin Centre for Climate Research, Stockholm University, Stockholm, Sweden; 7 School of Architecture, Civil and Environmental Engineering, École Polytechnique Fédérale de Lausanne, Lausanne, Switzerland

**Keywords:** Atmospheric science, Atmospheric chemistry

Correction to: *Nature Communications* 10.1038/s41467-020-18551-0, published online 1 October 2020.

The manuscript contains two instances in which chemical compounds are incorrectly presented. These are HSO_4−_ in Fig. 1b, which should read ${\rm{HSO}}_4^ -$, and HOIO2 in ref. ^85^, which should be HOIO_2_.

Furthermore, there should not be a hyphen between 30 and nm on page 6, line 2. In addition, there is inconsistent text alignment in the “Instrumentation and measurements” section in which full justified and left aligned text are mixed.

This has now been corrected in both the PDF and HTML versions of the Article.

